# ^31^P MRSI Coil Combination Using ^23^Na Sensitivity Information

**DOI:** 10.1002/mrm.70204

**Published:** 2025-11-27

**Authors:** Jiying Dai, Mark Gosselink, Zahra Shams, Martijn Froeling, Alexander Jan Eberhard Raaijmakers, Dennis Wilhelmus Johannes Klomp

**Affiliations:** ^1^ Precision Imaging Group University Medical Center Utrecht Utrecht Utrecht the Netherlands; ^2^ Tesla Dynamic Coils B.V. Zaltbommel Gelderland the Netherlands; ^3^ Medical Imaging Analysis Group, Biomedical Engineering Department Eindhoven University of Technology Eindhoven Noord‐Brabant the Netherlands

**Keywords:** MRSI, multi‐channel coil combination, multi‐nuclear

## Abstract

**Purpose:**

To optimally combine receive channels for ^31^P MRSI when the SNR is relatively low using sensitivity maps obtained from ^23^Na signals.

**Methods:**

This study utilized a dedicated X‐nuclei 15‐channel head coil array capable of imaging both ^23^Na and ^31^P. After acquiring the relatively low‐SNR multi‐channel ^31^P MRSI signals, also the high‐SNR ^23^Na sensitivity information was acquired. The low‐SNR ^31^P MRSI signals were combined using the high‐SNR ^23^Na sensitivity information, instead of the traditional self‐weighting approach where ^31^P sensitivity is derived from the low‐SNR ^31^P FID signals. The proposed method was evaluated by electromagnetic simulations, numerical Monte Carlo studies, and in vivo ^31^P MRSI on two healthy volunteers undergoing three independent acquisitions in total.

**Results:**

The electromagnetic simulations indicate a negligible SNR loss of maximum 5% by using ^23^Na sensitivities for ^31^P coil combination. The Monte Carlo synthetic coil combination shows that the higher SNR of the ^23^Na sensitivity information benefits the SNR of the combined ^31^P spectra. In addition, the self‐weighted method is prone to introducing combination bias (SNR overestimation), while the ^23^Na‐based method does not. The in vivo MR experiments demonstrate an increase in SNR when using the ^23^Na‐based combination, as verified across three independently acquired datasets.

**Conclusion:**

^31^P MRSI multi‐channel signal combination using ^23^Na sensitivities acquired with the same receiver array provides better performance when compared to self‐weighting.

## 1 Introduction

^31^P MRSI is a powerful tool for studies in cell proliferation and energy metabolism applied for instance in the monitoring of cancer treatment efficacy [1, 2, 3, 4, 5, 6]. Due to the low intrinsic sensitivity of ^31^P in the human body, like most X‐nuclear species when compared to ^1^H, multi‐channel local receivers are increasingly used [7, 8, 9, 10] primarily to boost the SNR [11]. However, the multi‐channel signal combination for ^31^P MRSI is not as straightforward as for ^1^H MRI. Suboptimal signal combination degrades the quality of spectroscopic data, failing to capitalize on the SNR efficiency enabled by advanced and often costly hardware innovations such as high‐field systems and phased‐array coils.

**FIGURE 1:**
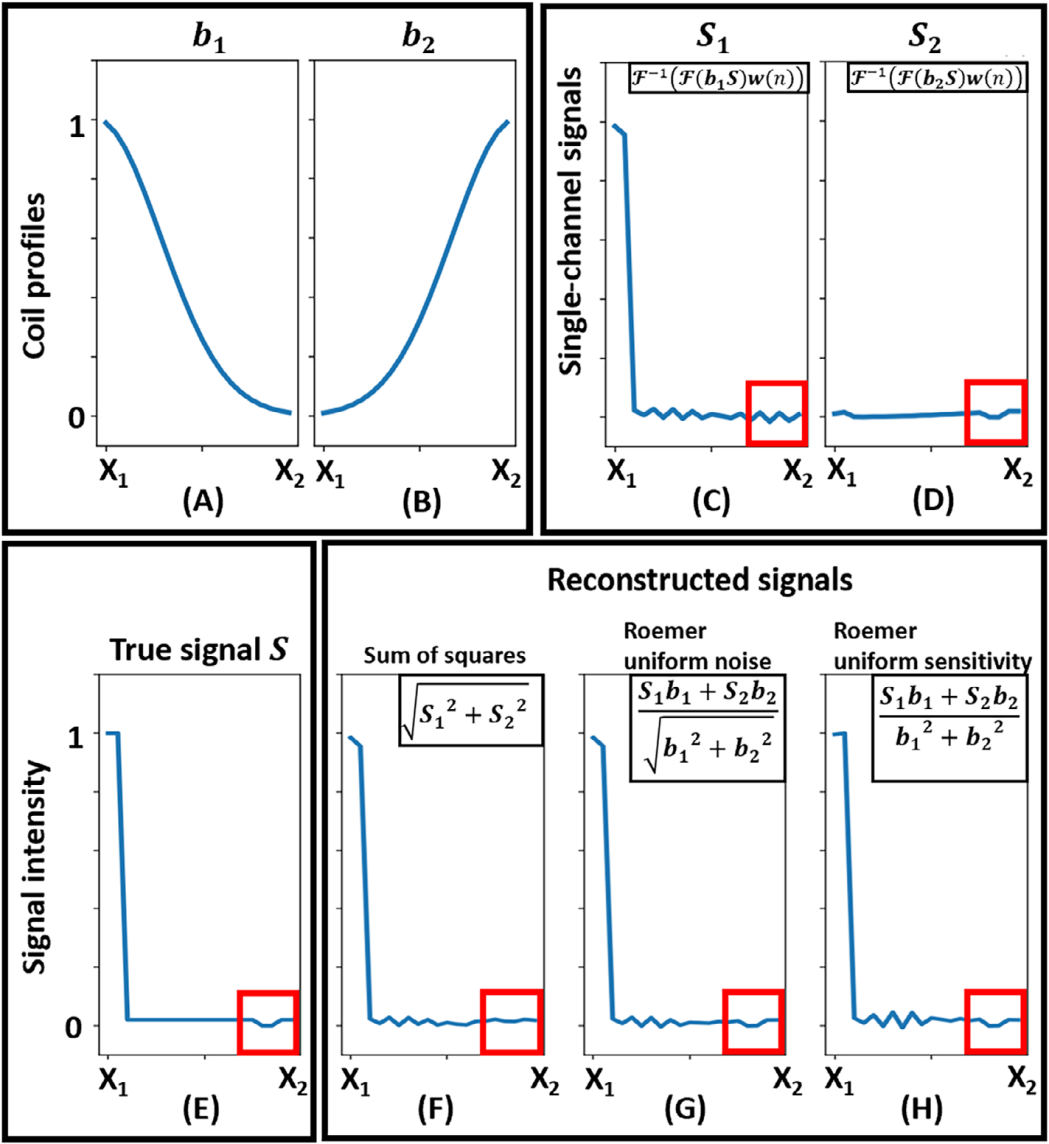
Schematic illustration of the point‐spread effect based on 1D numerical simulations. The model mimics a high signal from muscle near the skull, low signal from the brain, and zero signal from CSF. *X*_1_ and *X*_2_ denote the two ends of the FOV. (A, B): Coil sensitivity profiles of two synthetic receiver coils. (C, D) Corresponding received signals for each coil, assuming a truncation window w(n) that retains the central 95% of k‐space. (E) Ground truth signal. (F–H) Reconstructed signals using: (F) sum of squares; (G) Roemer uniform‐noise; (H) Roemer uniform‐sensitivity. Notably, a large ^31^P signal close to the coil (e.g., from muscle near the skull) can lead to substantial reconstruction errors in deeper regions (e.g., in CSF), as indicated by the red box.

For both ^1^H MRI and ^31^P MRSI, the optimal‐SNR coil combination method introduced by Roemer et al. [11] is widely used [12], where the coil sensitivity profiles determine the complex weighting factors in the coil combination. For ^1^H, coil sensitivity maps can be acquired rapidly due to the high SNR efficiency of ^1^H MRI. For ^31^P, direct acquisition of coil sensitivity maps is not possible within acceptable acquisition time due to low concentration of ^31^P in the human body. Therefore, the sensitivity maps are usually approximated from the ^31^P signals themselves, for example by taking the averages of the highest points of the FID signals or using singular value decomposition between the elements [13]. These methods work properly in most parts of the human brain or in muscles where the intrinsic SNR is sufficient. However, when the intrinsic SNR is low (e.g., cerebrospinal fluid in the brain, abdominal regions at depth, lungs), the approximated coil sensitivity profiles become less reliable, leading to further degradation of the combined SNR. Furthermore, voxel bleeding from neighboring regions, which is a common problem in low‐resolution MRSI, would dominate the weighting factors between the elements where the signal level drops sharply. When using the contaminated (i.e., by neighboring voxels) signals to approximate the coil profiles, the estimated coil profiles are contaminated. Consequently, the contamination of both the combined multi‐channel signals and the used coil sensitivity profiles amplifies the voxel bleeding effect even further. Figure 1 provides a brief illustration of this amplification effect based on a synthetic 1‐D 2‐channel dataset. The python code for the illustration is shared on GitHub [14].

There are a variety of approaches to determine the coil combination weights other than deriving from the received data. Rogers and Robson compared the 10 most‐used MRS combination methods [15] based on synthetic multi‐channel spectroscopy data (49.9 MHz). The tested methods determine the combination weights from three types of resources: simulated B_1_^−^, received data [13, 16, 17], reference scans or signals [18]. In their study, all tested methods can achieve above 90% of the optimal SNR when the intrinsic SNR is above 20. However, when the intrinsic SNR is below 8, the performance of all tested methods drops significantly (i.e., below 80% of the intrinsic SNR) except for the method using simulated B_1_^−^ maps generated by Biot‐Savart law. However, the advantage of using B_1_^−^ distributions simulated with Biot‐Savart law cannot be secured when the frequency goes higher (e.g., ^31^P at 7 T, which is 120.6 MHz) because Biot‐Savart law is based on quasi‐static assumptions which are not valid at higher frequencies. Moreover, none of these methods shows a distinct performance in terms of preventing the amplification of voxel bleeding.

In this paper, we investigated the feasibility of using ^23^Na sensitivity maps for ^31^P coil combination implementing a double‐tuned RF setup [19] that receives the ^23^Na and ^31^P signals with the same receiver array. A similar work was conducted by JD Sanchez‐Heredia et al. [20], where they combined ^13^C signals with ^23^Na sensitivity profiles at 3 T. The frequencies of ^13^C and ^23^Na are very close (i.e., 32.1 and 33.8 MHz at 3 T). As a result, a slight off‐tuning of the ^13^C coil can already cover both frequencies. Moreover, the low and the close frequencies make the wavelength difference within the subject almost negligible. When comparing ^31^P and ^23^Na at 7 T, their resonant frequencies are 120.7 and 78.9 MHz, which is further than ^13^C and ^23^Na at 3 T, but relatively close compared to most other nuclear species. On the other hand, the SNR of ^23^Na is significantly higher than that of ^31^P in the human body [21, 22, 23, 24]. For instance, in tumor tissue, MR detectable sodium concentrations can be up to 100 mM [25], whereas phosphocholine concentration can be as low as 1 mM [1]. Moreover, combining the gyromagnetic ratio and the spin number (i.e., 3/2 for ^23^Na and ½ for ^31^P), the magnetic moment of ^23^Na is about two times that of ^31^P, meaning a doubled magnetic signal intensity per nucleus [26]. In addition, in vivo ^23^Na typically has very low T_1_ (e.g., below 40 ms in human brain [25]), whereas ^31^P is well‐known for the opposite (i.e., above 1 s [27]). As a result, ^23^Na has a significantly higher SNR efficiency (i.e., SNR per unit time) compared to ^31^P for in vivo MR experiments. Therefore, through a quick acquisition of low‐resolution ^23^Na MRSI, or simply by approximating from the received ^23^Na signals, the coils' ^23^Na sensitivity maps can be derived. Using these ^23^Na sensitivity maps, we can apply the coil combination of the multi‐channel ^31^P MRSI signals that are acquired in the same scan session on the same subject.

We evaluated this combination method by electromagnetic (EM) simulations on the virtual human model DUKE [28], a Monte Carlo study on synthetic coil combination with added noise, and MR experiments on two healthy volunteers in three separate scans. The noise‐free EM simulations investigate the effect of the frequency difference between ^31^P (the to‐be‐combined signal) and ^23^Na (used for approximating sensitivity) on coil combination performance. The Monte Carlo study assesses the impact of noise, whether correlated or uncorrelated, present in the approximated sensitivity. Finally, the in vivo experiments integrate both aspects by examining their effects under realistic conditions. The in vivo validation comprises three separate scans: two repeated acquisitions on different healthy volunteers (Datasets 1 and 2), and a further acquisition with higher spatial resolution on one of the two volunteers (Datasets 3).

## 2 Methods

### 2.1 Hardware

**FIGURE 2:**
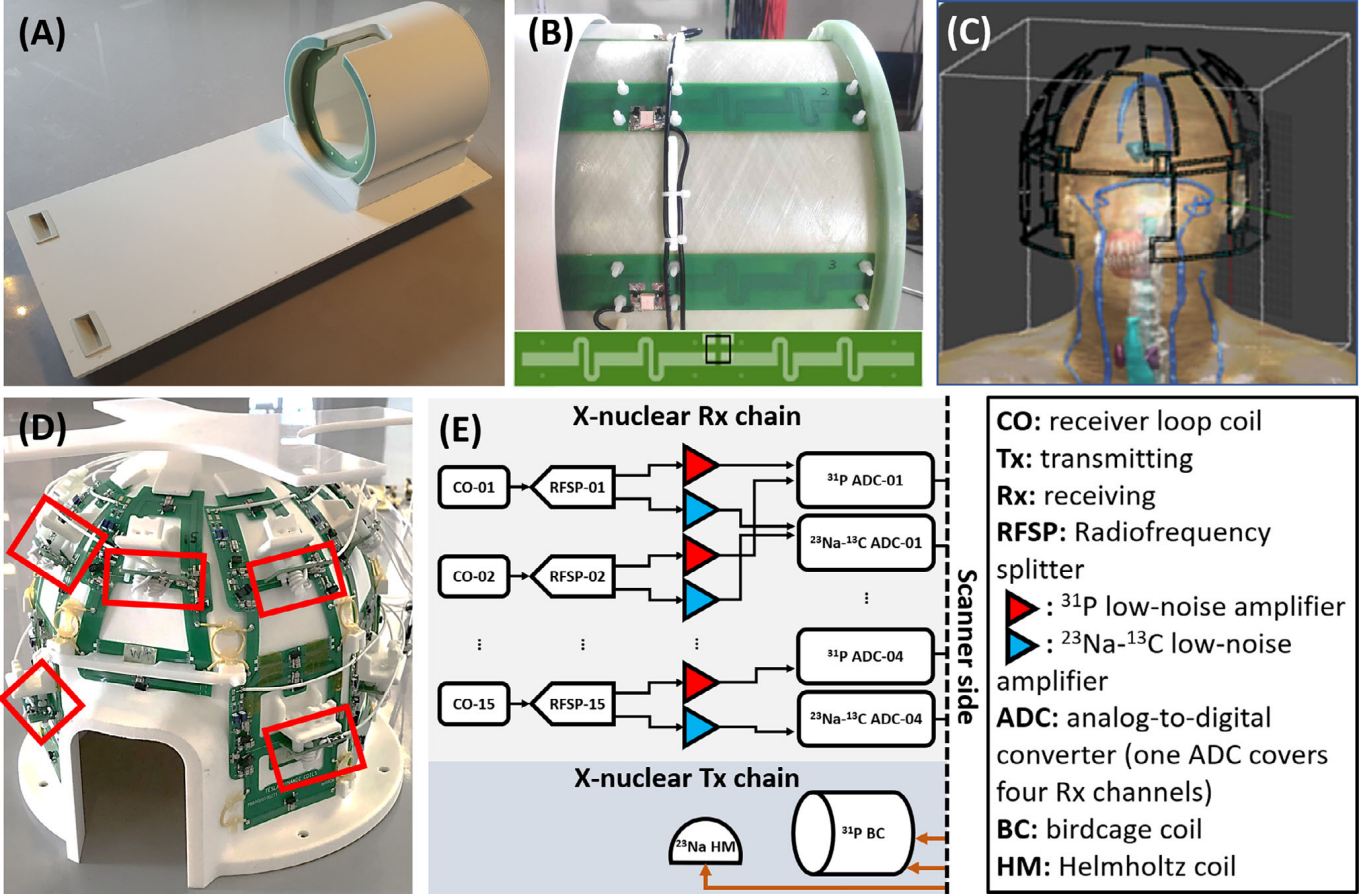
The used coil: (A) The complete coil. (B) Side view of the eight‐channel dipole array, with the dipole layout presented at the bottom. (C) The model for EM simulation. (D) The 15‐channel ^31^P‐^23^Na/^13^C receiver array of the coil. The red frames indicate the dual‐tuned cable traps (120.6 and 77 MHz). (E) The interfacing of the X‐nuclear part of the used coil.

We used a multi‐tuned RF head coil (META Brain coil, Tesla Dynamic Coils, Zaltbommel, the Netherlands) [19], that transmits and receives ^1^H B_1_ fields (298.0 MHz) with a cylinder‐mounted eight‐channel dipole array and receives B_1_ fields for ^31^P and ^23^Na (120.6 and 78.8 MHz) with a 15‐element surface loop array. The coil operates on a Philips 7 Tesla system (Philips Healthcare, Best, Netherlands). A birdcage coil embedded in the bore (Futura Composites, Heerhugowaard, the Netherlands) is used for ^31^P transmission [8]. A local Helmholtz coil is used for ^23^Na transmission. Figure 2 shows the coil setup. The detailed design including the coil circuit schematics can be found in our previous work [19]. Figure S2 shows Figure 2D at high resolution.

### 2.2 Simulation Models

The standard Roemer combination for spectroscopy data is self‐weighted, relying on coil sensitivity information approximated from the received spectroscopy signal. In this study, we aim to approximate sensitivity using the received ^23^Na signal instead. The primary differences between the sensitivity approximations derived from ^23^Na and ^31^P signals include: (a) the wavelength, (b) the SNR level, (c) the noise correlation with the received signal. To investigate the effects of these factors, we conducted two sets of simulations:

1. EM simulations: To evaluate how the wavelength difference between ^23^Na and ^31^P impacts the combination performance of the ^31^P signals.
2. Monte carlo study on synthetic coil combination: Using synthetic ^31^P spectroscopy data with added complex Gaussian noise, we compared performance across three scenarios: (a) using noise‐free sensitivity, (b) using the self‐weighted method (correlated noise), and (c) using sensitivity with independent noise (no noise correlation between sensitivity and MRSI data to be combined).

This section details the construction of simulation models.

#### 2.2.1 EM Simulations

EM simulations were performed using Sim4Life V7.2.1 (Zurich Med Tech, Switzerland) with the virtual human model DUKE [28]. We modeled the 15‐channel receiver array and simulated the B_1_^−^ fields for the frequencies of both ^31^P and ^23^Na at 7 T. The EM simulation model is shown in Figure 2C. The EM fields of each channel were exported with normalization to the currents on its corresponding loop.

#### 2.2.2 Synthetic Data for Coil Combination

We performed coil combination of synthetic spectroscopy data in MATLAB R2020a (MathWorks, Natick, MA) using a Monte Carlo method to illustrate how noise propagates during the combination process. This section describes how the spectroscopy data was synthesized.

We constructed a synthetic single‐voxel ^31^P spectrum, shown in Figure 6A; then applied inverse Fourier transform to generate the FID signal. Complex Gaussian noise was then introduced within the FID domain. The noise is denoted as follows:

$$n=\left(n_{1},\ldots ,n_{N_{\mathrm{ch}}}\right)$$

$$n_{k}∼N\left(0,\sigma^{2}I\right)+i*N\left(0,\sigma^{2}I\right),k=1,2,\ldots ,N_{\mathrm{ch}}$$

where $N_{\mathrm{ch}}$ represents the number of the receiver channels, $n_{k}$ has a dimension of $N_{\mathrm{FID}}\times 1$, where $N_{\mathrm{FID}}$ denotes the number of FID points. During the synthetic combination experiments (described later), σ was varied from low to high across three levels. The true coil sensitivity information is generated as a $1\times N_{\mathrm{ch}}$ vector $b_{\text{true}}$. The elements of $b_{\text{true}}$ mimic the identical amplitude and the quadrature phase from the receiver array:

$$b_{\text{true}}=\left[b_{\text{true},1},\ldots ,b_{\text{true},N_{\mathrm{ch}}}\right],b_{\text{true},k}=e^{\mathrm{ik}\frac{2\pi }{N_{\mathrm{ch}}}}.$$

As a result, we synthesized a $N_{\mathrm{ch}}$‐channel single‐voxel ^31^P MRS data p:

$$p=b_{\text{true}}s+n,$$

where s is the true ^31^P FID. There is no noise correlation between the channels. The combinations are repeated until the noise floor of the combined spectrum becomes negligible, meaning the combined spectrum is close to the expectancy.

### 2.3 MR Experiments

We performed MR experiments on two healthy volunteers. In total three datasets were acquired. For the first volunteer, two datasets were acquired using different spatial resolutions, with voxel sizes of 20 × 20 × 20 mm^3^ and 12 × 12 × 12 mm^3^, respectively. For the second volunteer, one dataset was acquired by repeating the 20 × 20 × 20 mm^3^ acquisition used for the first volunteer. The participants provided written informed consent, and the study was approved by the medical ethics committee of the University Medical Center Utrecht. The safety analysis of the transmit coils was included in the Investigation Medical Device Dossier (IMDD) and was also reviewed by the ethics committee. For the users' interest, we included in Supporting Information the SAR‐related constraints.

#### 2.3.1 MR Sequence

For the preparation of the in vivo X‐nuclear scan, we performed a ^1^H scout scan, followed by ^1^H‐based B_0_ mapping, B_0_ shimming, and another B_0_ mapping thereafter. Afterwards, we performed ^31^P CSI FID and ^23^Na CSI FID with the same acquisition grid (i.e., the same FOV and spatial resolution for the ^23^Na and ^31^P scans within one session). The subject location remains consistent during one scan session.

We applied two different resolutions to the CSI FID scans. The normal‐resolution ^31^P and ^23^Na scans have a FOV of 260(AP)×220(LR)×180(FH) mm^3^, with 20×20×20 mm^3^ voxel size. Further parameters are for ^31^P FA: 12°, TR: 60 ms, TE: 0.5 ms, spectral points: 256, readout bandwidth: 5000 Hz, Hamming weighted averaging with 70 averages of the center of k‐space, acquisition time: 11 min 42.7 s. For ^23^Na FA: 12°, TE: 0.55 ms, TR: 60 ms, 256 spectral points, 5000 Hz readout bandwidth, Hamming weighted averaging with 10 averages of the center of k‐space, acquisition time: 2 min 7.7 s. The high‐resolution ^31^P and ^23^Na scans have an FOV of 220(AP)×187(LR)×204(FH) mm^3^, with 12×12×12 mm^3^ voxel size. The ^31^P scan has 12° flip angle, 60 ms TR, 0.5 ms TE, 256 spectral points, 5000 Hz readout bandwidth, and Hamming weighted averaging with 20 averages of the center of k‐space, resulting in a total acquisition time of 17 min 22.9 s. The ^23^Na scan has a flip angle of 12°, 0.66 ms TE, 60 ms TR, 256 spectral points, 5000 Hz readout bandwidth, Hamming weighted averaging with 6 averages of the center of k‐space, resulting in a total acquisition time of 7 min 36.2 s. The flip angle of the ^31^P scans is set around the Ernst angle, as an average over the different T_1_ of ^31^P metabolites [27]. The ^23^Na scans employ a significantly more conservative flip angle to ensure safe usage of the experimental RF transmitter.

We also performed B_1_^+^ mapping for both ^31^P and ^23^Na on a homogeneous phantom to ensure sufficient B_1_^+^ homogeneity for both ^23^Na and ^31^P. Actual flip‐angle imaging (AFI) [29] was used for ^31^P B_1_^+^ mapping because ^31^P compounds have long T_1_. The double‐angle method (DAM) [30] was used for ^23^Na B_1_^+^ mapping considering the short T_1_ of ^23^Na.

#### 2.3.2 Global ^31^P‐ ^23^Na Phase Calibration

**FIGURE 3:**
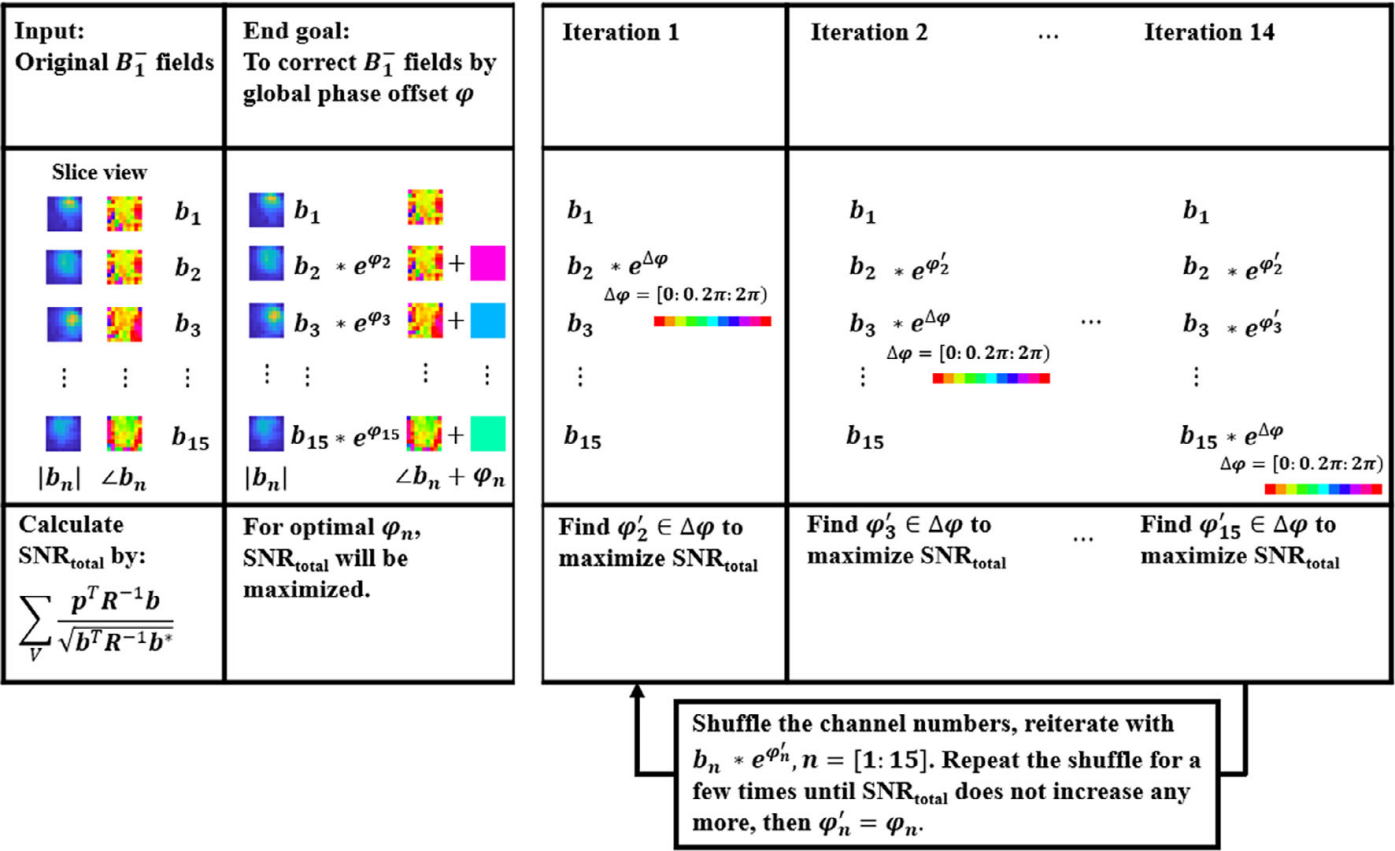
A flow chart description of the global phase correction process. $b_{n}$: ^23^Na sensitivity map ($B_{1}^{-}$) of channel, spatially dependent. p: Signal from channel n. ∆φ: Additional phase, no spatial dependence. ${\mathrm{SNR}}_{\text{total}}$: The whole‐volume SNR. $\varphi_{n}$: The channel‐wise non‐spatial dependent phase that maximizes ${\mathrm{SNR}}_{\text{total}}$. $\varphi_{n}^{\prime }$: Temporary candidates of $\varphi_{n}$, derived from the experienced iterations. R: n‐by‐n noise covariance matrix between channels. $\sum_{V}:$ Summation over the entire FOV.

Due to the hardware difference between the receive chains of ^23^Na and ^31^P (e.g., RF splitter, pi‐and‐tee network for preamp‐decoupling), there is a global phase offset between ^23^Na and ^31^P signals for every channel. This offset was determined numerically via an iterative optimization process where maximizing the whole‐volume SNR (i.e., summation of the SNR of all the voxels) is the optimization target. The optimization process consists of three steps: First, sweep the phase offset between the ^23^Na sensitivity maps of channels 1 and 2 (i.e., 0 to 2π, 0.2π increments). For every sweep value, perform ^31^P signal combination and calculate the optimization target. From this procedure, find out the optimal phase offset. Second, repeat the previous steps but now between channel 3 and the optimal summation of channel 1 and 2, and continue repeating until channel 15 is also included. Third, shuffle the channels' order randomly, and repeat the optimization until the whole‐volume SNR summation does not increase further after a few random shuffles. The optimization process is described in Figure 3.

### 2.4 Data Analysis

#### 2.4.1 EM Simulation Data Analysis

The EM simulations were exported and analyzed in Python using Spyder (Anaconda Inc., Austin, TX, USA). Afterwards, we performed ^31^P signal combination in Python using different sensitivity maps. We used the simulated ^31^P B_1_^−^ field distributions as an approximation of the ^31^P signals, then combined the approximated signals from 15 channels according to Roemer's optimal SNR method in uniform noise form as Equation (1), using the simulated B_1_^−^ of ^31^P and ^23^Na as the sensitivity maps respectively.

(1) $$P=C\frac{p^{T}R^{-1}b}{\sqrt{b^{T}R^{-1}b^{*}}}$$

P is the combined ^31^P signal. *C* is a constant that scales for display. p is in principle the received 15‐channel signal. However, in our simulation study, p is approximated by directly using the simulated 15‐channel ^31^P B_1_^−^. b is ideally the ^31^P coil sensitivity profile. In our case, we use the simulated ^23^Na B_1_^−^ as b to compute P, and compare that to when using ^31^P B_1_^−^ as b. R is the noise covariance matrix derived from the simulated electrical fields:

(2) $$R_{\mathrm{ik}}\equiv \int \sigma \left(x,y,z\right)E_{i}\left(x,y,z\right)E_{k}\left(x,y,z\right)\mathrm{dV}$$

σ is the conductivity. $E_{i}$ is the electrical fields of the ith receiver channel, with a unit current in the loop. x,y,z define the spatial coordinate. V is ideally the whole volume but in our case the simulated ROI of 30×30×30 cm^3^.

To evaluate the combined results, we calculate the SNR of the combined ^31^P signals according to Equation (3) [11], but translated into the vector form (Equation 4). The denotations in Equation (3) are identical to the referred work by Roemer et al. [11]. The combined signal with the exact ^31^P B_1_^−^ is referred to as having the optimal SNR.

(3) $${\mathrm{SNR}}^{2}=\frac{{\left(\mathrm{\omega MV}\right)}^{2}\sum_{i=1}^{N}\sum_{k=1}^{N}n_{i}n_{k}B_{\mathrm{ti}}B_{\mathrm{tk}}}{4\mathrm{kT}∆f\sum_{i=1}^{N}\sum_{k=1}^{N}n_{i}n_{k}R_{\mathrm{ik}}\cos\left(\theta_{i}-\theta_{k}\right)}$$

(4) $$\mathrm{SNR}=\frac{\mathrm{\omega MV}}{\sqrt{4\mathrm{kT}∆f}}\frac{p^{H}R^{-1}b\;}{\sqrt{b^{H}\;R^{-1}b}}$$

#### 2.4.2 Monte Carlo Study on Coil Combination With Synthetic Data

With the $N_{\mathrm{ch}}$‐channel synthetic spectroscopy data, knowing that the true sensitivity $b_{\text{true}}$ is not acquirable, we examined three methods of approximating the sensitivity b: the ideal method, the self‐weighted method, and the other‐weighted method (without noise correlation).

For the ideal method, b is approximated by taking the average of the first five FID points of the noise‐free signal. For the self‐weighted method, b is the average of the first five FID points of the noisy signal (i.e., the to‐be‐combined $N_{\mathrm{ch}}$‐channel signal). For the other‐weighted method, b is the average of the first five FID points of an $N_{\mathrm{ch}}$‐channel noisy signal which has no noise correlation to the to‐be‐combined signal, yet the noise variance is identical to the noisy signal. This experimental design aims to investigate the noise correlation factor independently of the sensitivity SNR level.

We apply the coil combination following Equation (1) in the FID domain. R is an $N_{\mathrm{ch}}\times N_{\mathrm{ch}}$ identity matrix as we assume no noise correlation between channels. Subsequently, we perform inverse Fourier transform to convert the combined FID signal into the spectral domain. Afterwards, the real part of the spectra from all Monte Carlo repetitions are averaged. A first‐order phase‐correction is applied at the end to maximize the real part of the PCr signal.

The MATLAB scripts for the Monte Carlo study are shared publicly [14].

#### 2.4.3 MR Experiment Data Analysis

The scan data were processed in MATLAB R2020a (MathWorks, Natick, MA). The sensitivity maps (B_1_^−^) of both ^31^P and ^23^Na were approximated by taking the averages of the second till the fifth points of the FID signals. Hamming filter was implemented standardly in the MRSI processing pipeline. We did not perform apodization. Roemer's uniform‐noise method (Equation 1) is used for signal combination. The variables in Equation (1) are slightly different from that when applied to the simulation results. p is the received multi‐channel ^31^P signal. R is the noise covariance matrix computed from the noise scan. b is the B_1_^−^ maps approximated from the received ^31^P signal or the received ^23^Na signal by taking the average of the second to the fifth FID data points.

Same as for the synthetic coil combination, the coil combination of the in vivo data was first done in the FID domain, followed by a Fourier transform for conversion to the spectral domain. First‐order and zero‐order phase corrections were applied at the final stage.

For SNR evaluation, the standard deviation of the real part of the spectrum within the 10–20 ppm range was used as the noise. Two signal definitions were employed: one is based on the peak height of the real part of the PCr signal and the other on the α‐ATP signal. The resulting SNR values are referred to as SNR_PCr_ and SNR_α‐ATP_ respectively.

## 3 Results

### 3.1 Simulations

#### 3.1.1 EM Simulations

**FIGURE 4:**
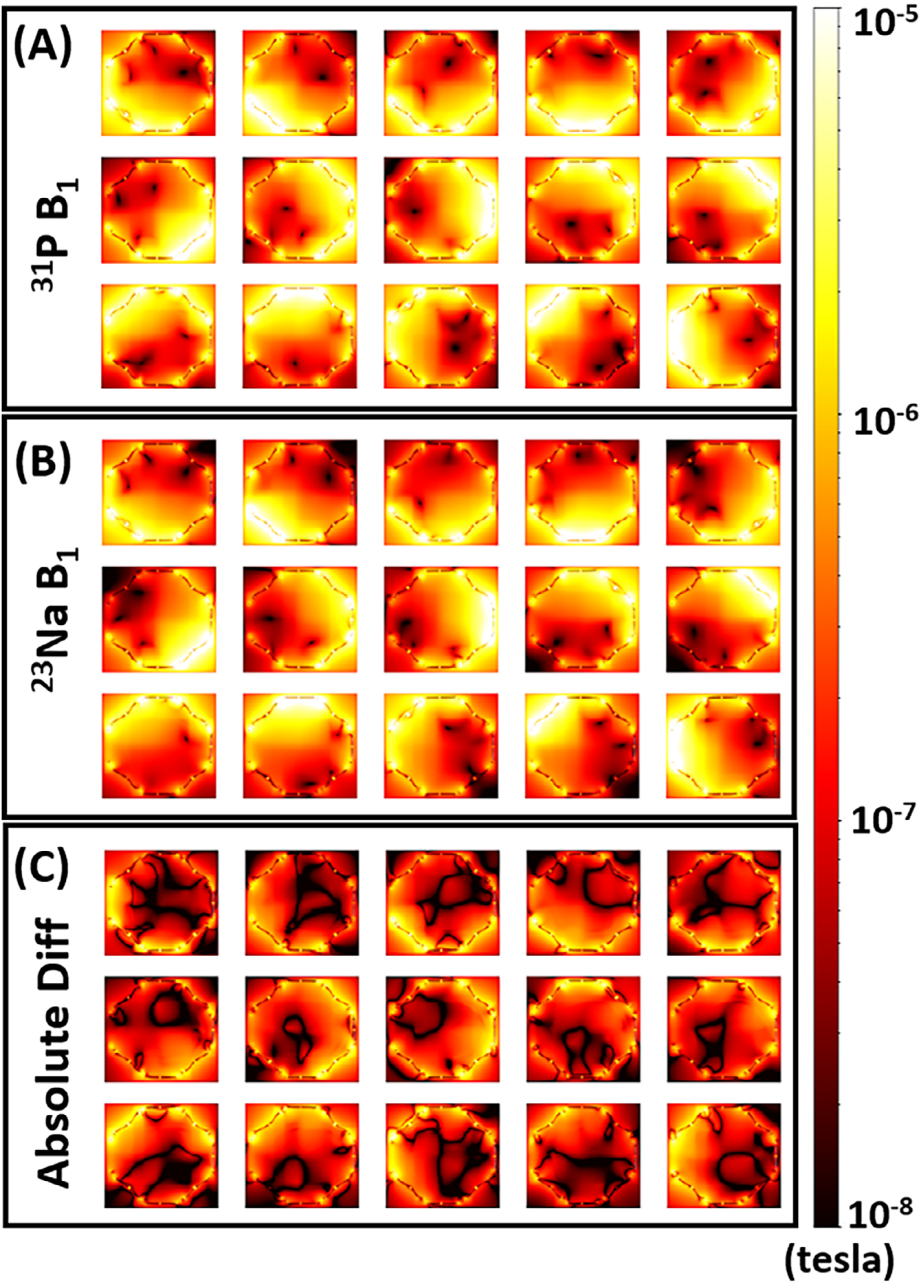
EM simulations of all 15 channels, presented in logarithm color scale: (A) B_1_^−^ at 120.6 MHz (^31^P at 7 T); (B) B_1_^−^ at 78.9 MHz (^23^Na at 7 T); (C) The absolute difference between A and B.

Figure 4A,B shows the simulated B_1_^−^ maps (center slice) of ^23^Na and ^31^P at 7 T. We use a logarithmic color scale for better display. The B_1_^−^ maps at these two frequencies show high similarity. Figure 4C shows the difference between the ^23^Na B_1_^−^ and the ^31^P B_1_^−^distributions.

**FIGURE 5:**
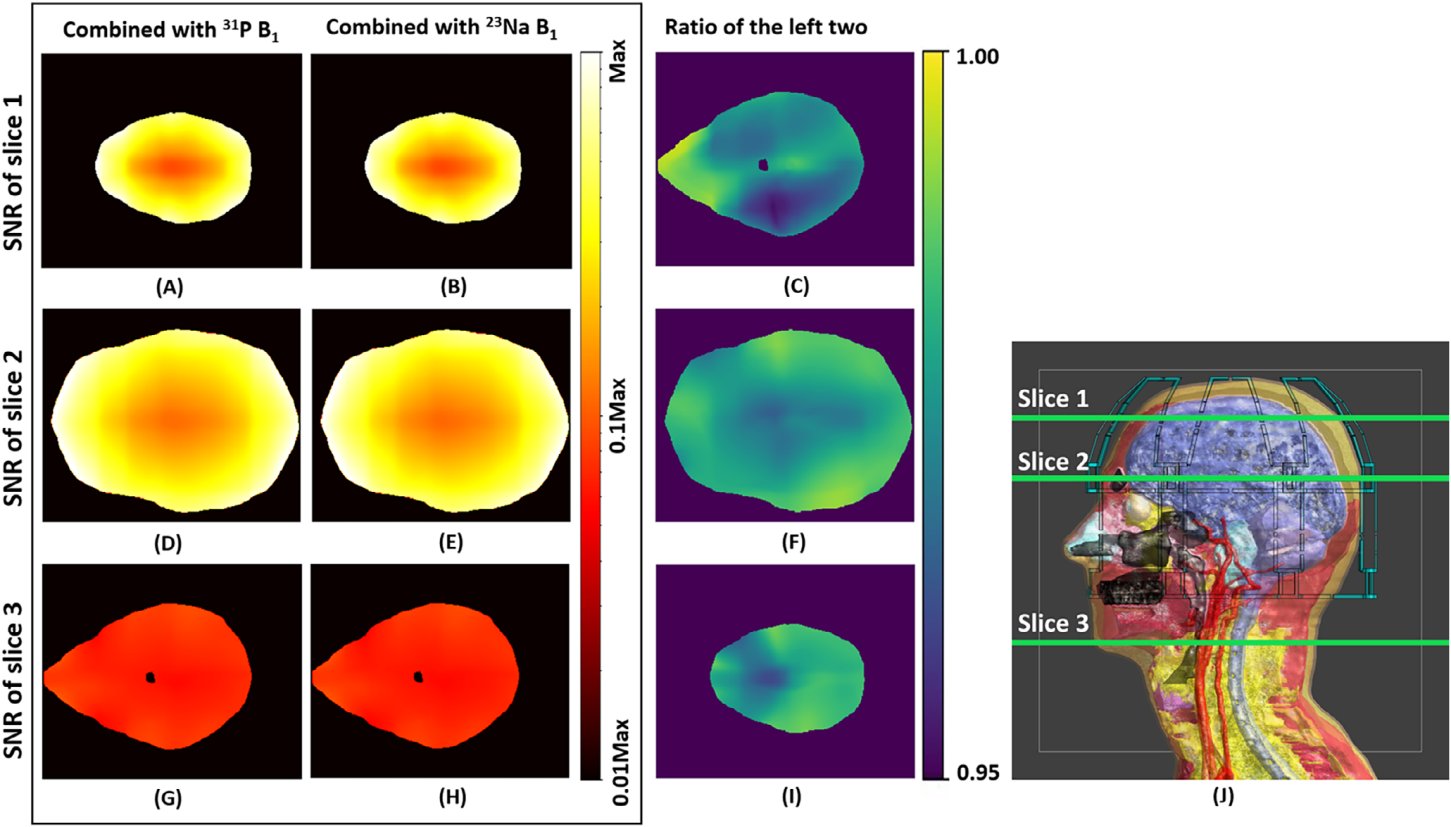
Simulated SNR maps of the combined ^31^P signals, over the central transverse slice within Duke's head and slices from two ends of the head: (A, D, G) using ^31^P B_1_^−^, (B, E, H) using ^23^Na B_1_^−^. (C, F, I) Ratio of SNR combined with ^23^Na over SNR combined with ^31^P. (J) Indication of slices in the sagittal view.

In addition to the qualitative and quantitative comparison between the B_1_^−^ fields of ^23^Na and ^31^P, we further performed SNR evaluations of signal combinations using either of these two types of B_1_^−^ maps to make a more comprehensive comparison: Figure 5 shows three transverse slices of the SNR maps of the combined ^31^P signal using ^31^P B_1_^−^ and ^23^Na B_1_^−^ respectively. To better visualize subtle differences, the third column of Figure 5 includes the ratio between the SNR of the combined ^31^P signal using ^23^Na B_1_^−^ and using ^31^P B_1_^−^. The ratio shows that the SNR by using ^23^Na B_1_^−^ for combination is about 97%–98% of the SNR using ^31^P B_1_^−^ for combination. The minimum ratio is about 95%, meaning a maximum of 5% SNR is lost when using the sensitivity from ^23^Na as compared to the theoretical sensitivity from ^31^P.

#### 3.1.2 Synthetic Coil Combinations

**FIGURE 6:**
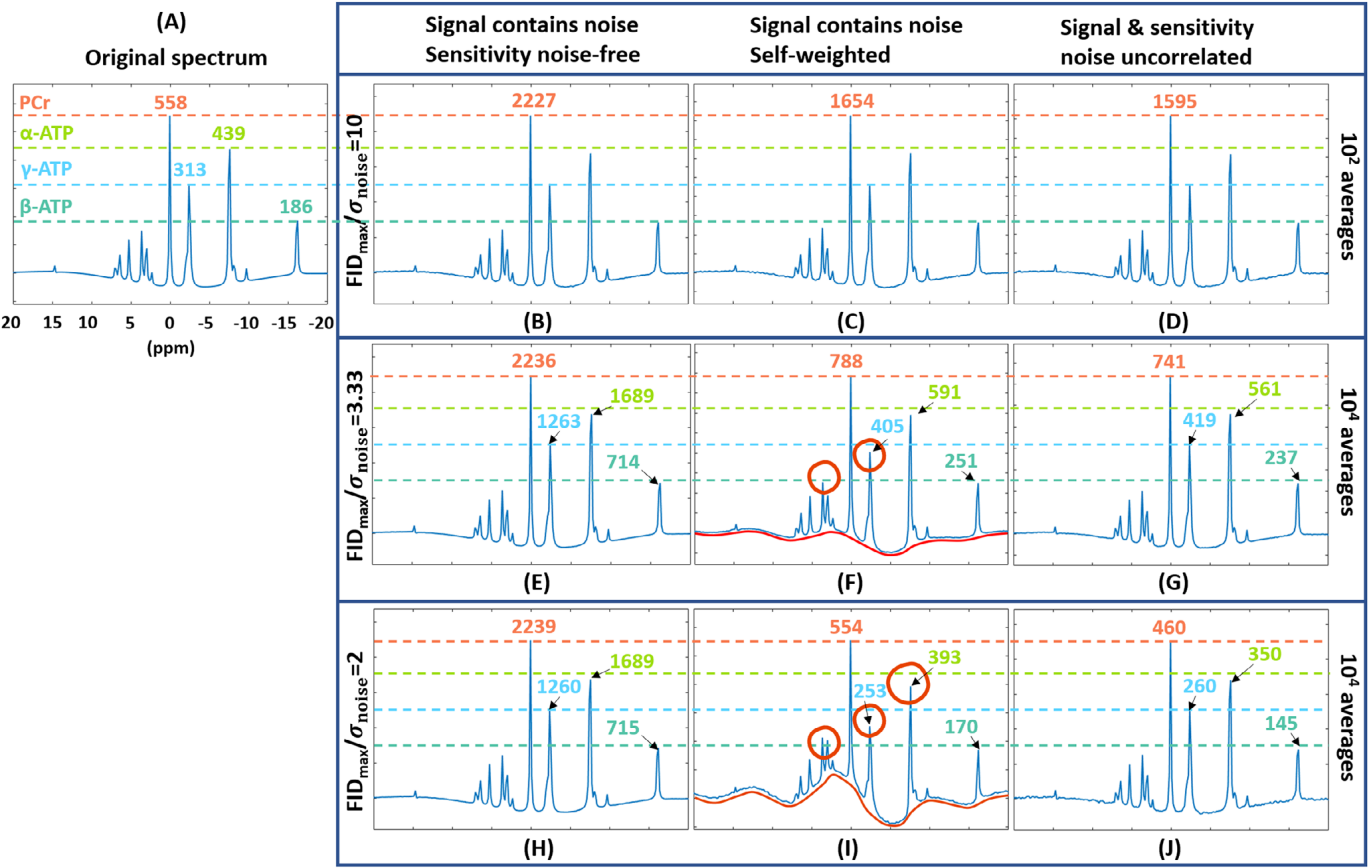
Expectations of 16‐Channel coil‐combination based on synthetic single‐voxel ^31^P MRS data using Monte Carlo method. (A) The true spectrum; (B–D) The combined spectrum using 16‐channel data with FID_max_/*σ*_noise_ = 10 per channel, combined using respectively (B) noise‐free sensitivity information, (C) sensitivity information approximated from the average of the first five FID points of the received signal, (D) same as (C) except the “received signal” used for sensitivity approximation has uncorrelated noise from the real received signal (mimicking a separately acquired data). Results shown in (B–D) are averaged from 10^2^ repetitions. (E–G) Similar to (B–D) but with FID_max_/*σ*_noise_ = 3.33 per channel, and the shown results are averaged from 10^4^ repetitions. (H–J) Similar to (B–D) and (E–G) but with FID_max_/*σ*_noise_ = 2 per channel. The colored dashed lines are to mark references to compare the height of the spectral peaks relatively. The spectra are displayed with a normalization to the PCr signal. The colored numbers (a.u.) are added to quantify the peak heights.

The synthetic coil combination results of 16 equally contributing receiver channels and three different SNR levels are shown in Figure 6B–J. The spectra are normalized to the PCr peak. For Figure 6B–D, the variance of the added noise per channel was defined as 10% of the amplitude of the maximum of the true FID signal (i.e., FID_max_/*σ*_noise_ = 10), and the combinations were repeated for 10^2^ times for averaging. For Figure 6E–G, the variance of the added noise per channel was defined as 30% of the amplitude of the maximum of the true FID signal (i.e., FID_max_/*σ*_noise_ = 3.33), leading to 10^4^ repetitions to end up with the shown spectra. For Figure 6H–J, the variance of the added noise per channel is 50% of the amplitude of the maximum of the true FID signal (i.e., FID_max_/*σ*_noise_ = 2), also 10^4^ repetitions applied. The ideal sensitivity leads to a perfect combination for all three SNR levels, showing no distortion and a peak height of about 4 (i.e., $\sqrt{N_{\mathrm{ch}}}$) times the true signal as expected. With a high SNR level (Figure 6B–D), all three methods show good performance. When the SNR is relatively low, the self‐weighted Roemer combination leads to a distorted baseline, as well as an irregular error over the spectrum, marked in red. The red circles in Figure 6F,I highlight the erroneous estimation of the phosphate peak amplitudes. The other‐weighted method does not exhibit such distortion. As the noise level of the combination results does not vary for different methods, the peak height of the combined spectra can be seen as an indication of the apparent SNR level. The self‐weighted method always leads to a slightly higher peak height compared to the other‐weighted method, indicating a slightly higher apparent SNR. On the other hand, the noise‐free sensitivity always leads to a combination of the highest true SNR, meaning the SNR level of the sensitivity information does affect that of the combined result.

### 3.2 B_1_ ^+^ Verifications

**FIGURE 7:**
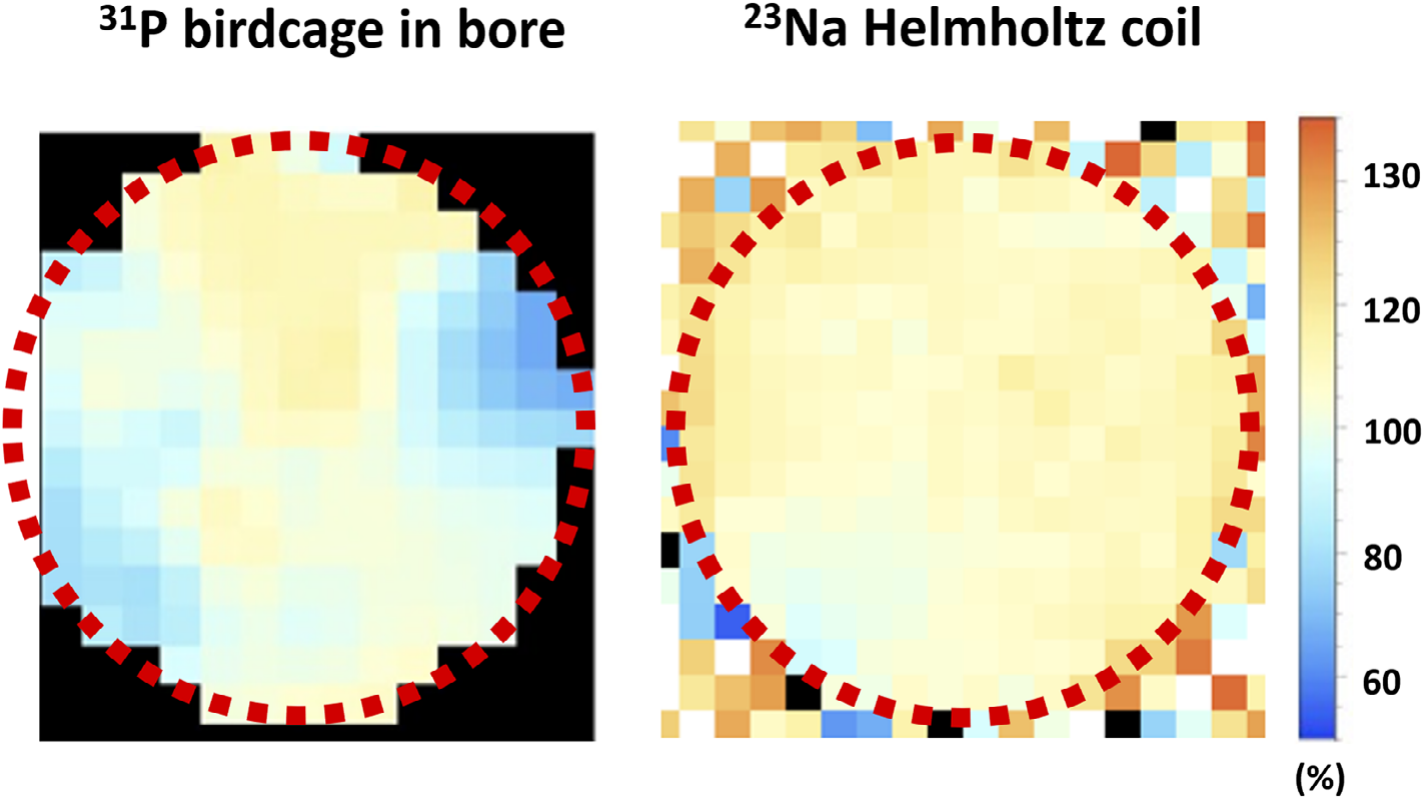
B_1_^+^ fields of the ^31^P birdcage bore coil (acquired by AFI [29]) and the Helmholtz ^23^Na coil (acquired by DAM [30]). The central slice of a 17‐cm‐diameter spherical phantom is shown. The phantom area is marked by the red dashed‐line circle. The resolution is 1 × 1 × 1 cm^3^. The unit is percentage of the nominal flip angle.

We performed B_1_^+^ mapping of both ^23^Na and ^31^P to verify there is no signal void caused by lack of excitation. Figure 7 shows the central‐slice B_1_^+^ amplitude map excited by the ^31^P birdcage coil and the ^23^Na Helmholtz coil on a spherical ^23^Na‐^31^P phantom with a diameter of 17 cm. The resolution is 1 × 1 × 1 cm^3^. The unit is the percentage of the nominal flip angle. The ^23^Na B_1_^+^ amplitude shows good homogeneity with less than 15% variation within the central slice. The ^31^P B_1_^+^ amplitude shows a non‐negligible spatial variation of over 30%, yet no B_1_^+^ void that would prohibit obtaining the B_1_^−^ maps from the MRI data.

### 3.3 In Vivo Experiments

**FIGURE 8:**
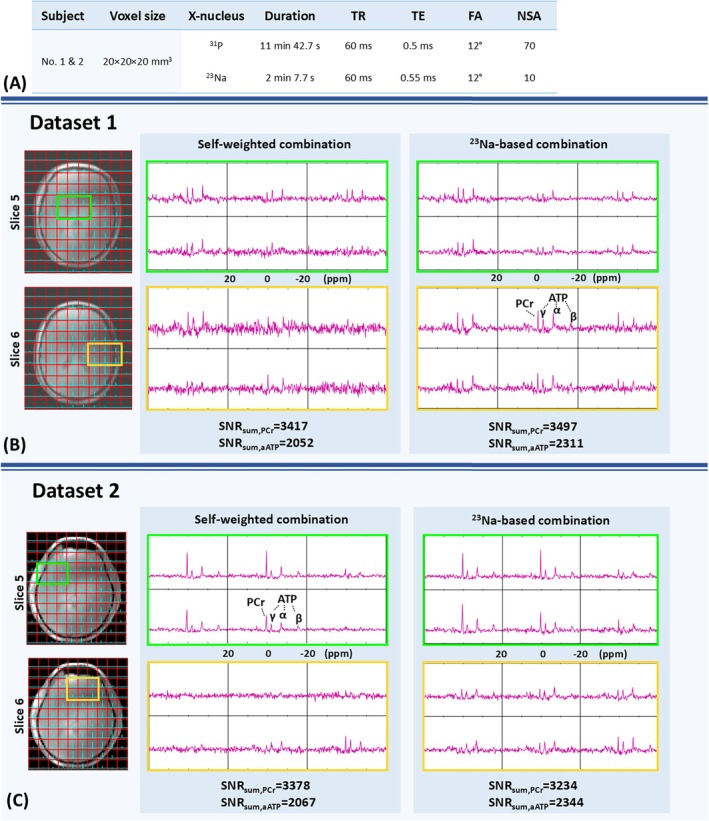
MR experiment results on two healthy volunteers (Dataset 1 and 2). (A) Corresponding scan parameters. (B) Two transverse slices from Dataset 1. On the left, the ^23^Na CSI FID (cyan) overlays on anatomical images. On the right, the ^31^P CSI FID (magenta) of the highlighted voxels is shown, combined using two different sensitivity approximation methods. The spectral range spans from −20 to 20 ppm (right to left). At the bottom, the resulting whole‐volume SNRs for both methods are shown individually. (C) Same as (B), but applied to Dataset 2, representing a repeated scan on a second healthy volunteer.

**FIGURE 9:**
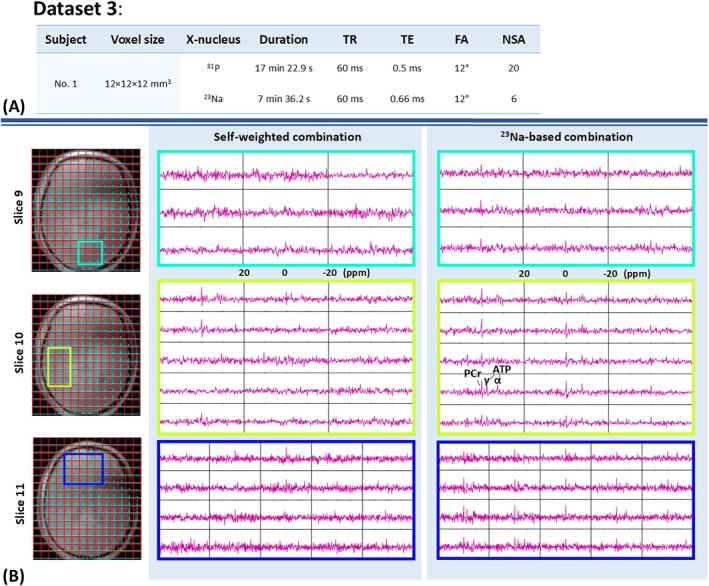
High‐resolution MR experiment results on a healthy volunteer (Dataset 3). (A) Corresponding scan parameters. (B) Two transverse slices from Dataset 3. On the left, the ^23^Na CSI FID (cyan) overlays on anatomical images. On the right, the ^31^P CSI FID (magenta) of the highlighted voxels is shown, combined using two different sensitivity approximation methods. The spectral range spans from −20 to 20 ppm (right to left).

Figures 8 and 9 show the combined ^31^P MRSI of the in vivo experiments. Figure 8B,C show two transverse slices of the combined CSI FID of Dataset 1 and 2, corresponding to scans acquired from two different volunteers using the same 20 × 20 × 20 mm^3^ resolution. On the left, the ^23^Na CSI FID is overlaid on anatomical images. On the right, the ^31^P CSI FID of the highlighted voxels is shown, combined using two different sensitivity approximation methods. At the bottom, the resulting whole‐volume SNRs for both methods are shown individually. Figure 9 presents three transverse slices of the combined CSI FID from Dataset 3, corresponding to the 12 × 12 × 12 mm^3^ resolution scan on the first volunteer, following the same layout as Figure 8.

The ^23^Na‐based method consistently demonstrates a higher SNR, reflected in improved visibility of the spectral peaks. The enhancement in spectral quality is most evident in the highlighted voxels of slice 6 in Datasets 1 and 2, as well as slices 10 and 11 in Dataset 3. This comparison can be quantified by comparing the SNR summation over the whole FOV. When comparing the PCr‐based SNR summation, the ^23^Na‐based method is very close to the self‐weighted method. When comparing the α‐ATP‐based SNR, the ^23^Na‐based combination achieves 13% higher SNR than the self‐weighted combination, for both Dataset 1 and 2. Most voxels in Dataset 3 lack sufficient “signal” for SNR calculation due to the high resolution and fewer averages. As a result, whole‐volume SNR quantification was not applied to Dataset 3.

## 4 Discussion

We evaluated the feasibility of combining multi‐channel ^31^P acquisitions using the B_1_^−^ distributions approximated from the ^23^Na signal received by the same multi‐tuned receiver array. The results were compared with the traditional combination method, which is to approximate the B_1_^−^ distributions from the received ^31^P signal.

The evaluation by EM simulations shows that the SNR loss by using ^23^Na B_1_^−^ to combine ^31^P signals is not more than 5%, despite the about 42 MHz Larmor frequency offset between these two nuclear species. Based on this, we can conclude that the difference in frequency does not cause significant SNR degradation to the combined results.

On the other hand, the Monte Carlo synthetic coil combination study shows that, the self‐weighted method leads to a biased expectancy when the SNR is low, appearing as a distorted spectrum baseline and a bias that is inconsistent over the spectral axis. This distortion becomes more pronounced as the SNR decreases. The case with FID_max_/*σ*_noise_ = 3.33 (i.e., Figure 6E–G) is considered as a low SNR but not even the worst SNR according to authors' experience. This distortion arises from the complex noise present in the received multi‐channel signal. When using self‐weighted Roemer combination, the noise correlation between the signal and the sensitivity information (i.e., b and p in Equation 1) leads to a constructive combination of the noise term that translates to an SNR‐dependent over‐estimation of the combined FID signal. This type of over‐estimation has been reported for the magnitude image of conventional MRI or when using root‐sum‐of‐squares for coil combination [31, 32, 33]. However, this issue is usually not significant for conventional MRI as ^1^H SNR is tremendously higher compared to that for ^31^P, and the ^1^H sensitivity information can be easily and separately acquired. For spectroscopy, the SNR is significantly lower by a factor greater than 1000 and the sensitivity cannot be acquired within a reasonable time. When using self‐weighted Roemer method, the combined result is similar to root‐sum‐of‐squares and follows a non‐central chi distribution resulting in an over‐estimation of the true signal. The over‐estimation scales up with the number of channels, as the SNR decreases, and when taking more FID points' average for sensitivity approximation (meaning more noise correlation between the signal and the sensitivity). Moreover, the signal combination is performed in the FID domain. After converting the combined FID signal into the spectral domain through a Fourier transform, this SNR‐dependent over‐estimation sums up along the FID axis with variable phases, leading to an over‐estimation that is not only SNR‐dependent, but also frequency/spectral‐dependent. According to the Monte Carlo study, the worst over‐estimation appears near the central frequency (i.e., 0–5 ppm). In our examples, the PCr peak is always centered in the readout bandwidth, thus the PCr peak has worse over‐estimation compared to for example the α‐ATP peak.

In contrary to the self‐weighted method, the ^23^Na‐based method uses independently acquired sensitivity information (from the ^23^Na scan). Therefore, the over‐estimation or the bias in the expectancy does not exist. This also explains why the self‐weighted method shows slightly higher peak than the other‐weighted method, when the two different sensitivities have the same noise level. When the SNR of the sensitivity increases, the SNR of the combined result also increases, as shown in Figure 6B,E,H. This is because the weight vector $\frac{R^{-\frac{1}{2}}\;b\;}{\sqrt{b^{H}\;R^{-1}b}}$ follows Kent distribution, and its norm decreases as the SNR of the used sensitivity b decreases (Figure S5). In realistic scenarios, ^23^Na concentrations are an order of magnitude higher than ^31^P concentrations in most biomedical applications, securing a higher‐SNR sensitivity information than the ^31^P‐based method.

Before analyzing the in vivo data, the B_1_^+^ mapping of both ^23^Na and ^31^P proves there is no excitation void at both frequencies within the FOV. This concludes that the parameter B_1_^+^ can be excluded from the comparison of the combination methods. To be specific, the $\frac{1}{\sqrt{b^{H}\;R^{-1}b}}$ term of Equation (1) divides the contrast information out of the combination weights $\frac{R^{-\frac{1}{2}}\;b\;}{\sqrt{b^{H}\;R^{-1}b}}$. Therefore, as long as b is not nulled by a void in B_1_^+^, the comparison between the combination methods is constrained within the scope of interest, that is the quality of the approximated sensitivity information b, in two dimensions: (1) The SNR of b, determined by the SNR of the signal used for sensitivity approximation (e.g., ^23^Na is high, ^31^P is low); (2) The accuracy of b, which can be degraded by the wavelength offset between the exact sensitivity and the approximated sensitivity (i.e., ^23^Na to ^31^P) or the noise correlation between b and p (when using self‐weighted combination).

The theory of SNR gain using the ^23^Na‐based method is supported by the in vivo experiments, with consistent improvements observed across all three datasets. According to the quantitative SNR analysis, the ^23^Na‐based combination yields a higher α‐ATP‐based SNR. This advantage is less pronounced when the SNR is calculated based on the PCr signal. Given that the PCr peak, located at the center of the readout bandwidth, is more susceptible to noise‐induced overestimation than the α‐ATP peak, this explains why the self‐weighted method appears to perform “better” when the SNR is calculated based on the PCr peak. We also present in Supporting Information the whole‐volume SNR_PCr_ and SNR_α‐ATP_ for both combination methods, using sensitivities approximated from the average of more (i.e., the 2nd to the 50th) FID data points. Figure S1 shows the corresponding SNR comparison for Dataset 1 and 2. This analysis shows how the SNR overestimation overrides when the noise correlation between the received signal and the approximated sensitivity increases. Figure S3 presents a particularly extreme scenario, in which the average of all 256 FID points are used for sensitivity approximation, leading to a combined SNR that significantly exceeds the theoretical upper limit. Figure S3, along with Section 4 of the Supporting Information, also includes extensive Monte Carlo‐based analyses, such as an evaluation of the potential limitations of the ^23^Na‐based method. Section 5 of the Supporting Information presents a phantom verification performed using the scan parameters listed in Table S1. The results of the phantom experiment (Figure S4) closely resemble the estimates from the Monte Carlo simulation (Figure S3).

Both simulations and the in vivo experiments show that the ^23^Na‐based combination has great potential to combine ^31^P signals. The high SNR of the ^23^Na signal secures high‐SNR sensitivity distributions. The absence of noise correlation between the sensitivity and the signal avoids the noise‐induced expectancy bias. The SNR of the ^31^P‐based sensitivity can be improved by taking the averages from more FID points. However, it will lead to a stronger correlation between the signal and the sensitivity. Consequently, when looking at a “better” SNR performance, it would be hard to tell if it is a benefit of the higher‐SNR sensitivity, or worse erroneous overestimation. For the same reason, we cannot draw any conclusion regarding the performance in terms of the extra point‐spreading effect.

In this study, we compared the ^23^Na‐based Roemer combination method to the ^31^P‐based Roemer combination for ^31^P MRSI. The ^23^Na‐based approach demonstrates significant improvements in SNR and completely avoids the SNR‐ and spectral‐dependent erroneous overestimation inherently present in the self‐weighted method. Compared to the methods that generate the coil combination weights from a reference scan or a reference signal, the ^23^Na‐based coil combination has the advantage that it does not require an additional ^31^P scan. Compared to the methods that derive the combination weights from the received signals, such as WSVD [13] or Brown's method [16], the ^23^Na‐based combination method has the advantage of being independent from the data quality. Moreover, any possible constructive add‐up of the correlated noise between the sensitivity and the signal is prevented, which eliminates erroneous overestimation of the combined signals.

An enabling factor of the ^23^Na‐based Roemer method is that it relies on a dedicated multi‐tuned receiver array. Our previous study demonstrated that the SNR loss of a multiple‐tuned RF coil can be limited to 15%–30% for the X‐nuclear species [19], while covering five nuclear species including ^23^Na and ^31^P. The availability of ^23^Na MRI can potentially also provide benefits to tissues where sodium is considered a biomarker for disease stage. ^23^Na yields the highest MR signal beside ^1^H in the human body. In addition to the versatility of ^23^Na MRI itself [34, 35, 36, 37], and providing B_1_^−^ estimations for coil combination attributing to its intermediate Larmor frequency among most of the MR‐sensitive nuclear species, a swift ^23^Na MRI can also be considered for accelerating other‐nuclei‐based MRI or MRSI [38].

## 5 Conclusion

We have presented a feasibility study of ^31^P multi‐channel signal combination using cross‐frequency B_1_^−^ at the ^23^Na Larmor frequency of the same receiver array. We have demonstrated a promising upper boundary by EM simulations, the capability of preventing an erroneous overestimation by Monte Carlo simulations, and we verified the SNR advantage by MR experiments. We believe that signal combination based on cross‐frequency receiver coils would bring us a step forward toward quantitative ^31^P MRSI, particularly in tissues with very low SNR (i.e., lungs [39]). Furthermore, this concept can be propagated to other multi‐tuned coils, such as ^1^H‐^19^F, ^2^H to ^17^O.

## Funding

This work was supported by NWO and KWF (17907), EU‐EIC‐Transition MITI (101058229), EU‐IHI‐Illuminate (101172722).

## Disclosure

Funded by the European Union, the private members, and those contributing partners of the IHI JU. Views and opinions expressed are however those of the author(s) only and do not necessarily reflect those of the member parties or contributing partners. Neither of the member parties nor contributing partners can be held responsible for them.

## Conflicts of Interest

Jiying Dai is also employed by Tesla Dynamic Coils B.V. as an RF engineer.

## Supporting information


**Figure S1:** The whole‐volume SNR results for Datasets 1 and 2 are shown, comparing three coil combination methods: ^31^P (self)‐weighted, ^23^Na‐weighted, and denoised‐^31^P‐weighted. For each method, SNR values are presented using sensitivities approximated from the 2nd to 5th FID points and from the 2nd to 50th FID points, respectively. SNR is calculated in two ways: based on the PCr signal and based on the α‐ATP signal.
**Figure S2:** High‐resolution picture of the 16‐channel ^31^P‐^23^Na/^13^C receiver loop array of the used head coil.
**Figure S3:** Monte Carlo‐based SNR estimations performed for the 15‐channel coil combination using both the self‐weighted and the ^23^Na‐based methods, across a range of intrinsic total SNR values. Additionally, a self‐weighted method using the full FID (all 256 FID points) average for sensitivity approximation is included, demonstrating the extent of overestimation in this extreme scenario. The “Perfect combination” represents the theoretical upper limit of the combined SNR; any value above it indicates an overestimation bias. The SNR was evaluated based on the PCr peak (A) and the α‐ATP peak (B), respectively. The synthetic ^31^P spectrum shown in Figure 6A was used as the input. The SNR ratio between the ^23^Na data (used for sensitivity approximation) and the ^31^P data was aligned with values observed in the presented in vivo datasets. For SNR calculation, the signal was defined as the mean over 3000 repetitions, while the noise was estimated as the standard deviation of the spectral noise floor, specifically within the 10–13 ppm and 15–20 ppm ranges.
**Figure S4:** Scatter plot of the combined SNR for all voxels within the spherical phantom, comparing the self‐weighted method (red) and the ^23^Na‐based method (blue). SNR_ref_ represents the combined SNR obtained using the denoised ^31^P multi‐channel signal as the sensitivity reference.
**Figure S5:** Monte Carlo simulation of a 16‐channel receiver array with randomized phases. The curve shows the $L^{1}$ norm of the weight vector as a function of per‐channel SNR (0–22), averaged over 10^4^ repetitions.
**Table S1:** Scan parameters of the phantom experiment.

## Data Availability

The data that support the findings of this study are available on request from the corresponding author. The data are not publicly available due to privacy or ethical restrictions.
