# Supplementary material for: 31P MRSI Coil Combination Using 23Na Sensitivity Information
Source: Magn Reson Med. 2025 Nov 27;95(4):1907–20. doi: 10.1002/mrm.70204 (PMC12850625; doi:10.1002/mrm.70204)
Supplement: Supplementary file 1 — Figure S1: The whole‐volume SNR results for Datasets 1 and 2 are shown, comparing three coil combination methods: 31P (self)‐weighted, 23Na‐weighted, and denoised‐31P‐weighted. For each method, SNR values are presented using sensitivities approximated from the 2nd to 5th FID points and from the 2nd to 50th FID points, respectively. SNR is calculated in two ways: based on the PCr signal and based on the α‐ATP signal. Figure S2: High‐resolution picture of the 16‐channel 31P‐23Na/13C receiver loop array of the used head coil. Figure S3: Monte Carlo‐based SNR estimations performed for the 15‐channel coil combination using both the self‐weighted and the 23Na‐based methods, across a range of intrinsic total SNR values. Additionally, a self‐weighted method using the full FID (all 256 FID points) average for sensitivity approximation is included, demonstrating the extent of overestimation in this extreme scenario. The “Perfect combination” represents the theoretical upper limit of the combined SNR; any value above it indicates an overestimation bias. The SNR was evaluated based on the PCr peak (A) and the α‐ATP peak (B), respectively. The synthetic 31P spectrum shown in Figure 6A was used as the input. The SNR ratio between the 23Na data (used for sensitivity approximation) and the 31P data was aligned with values observed in the presented in vivo datasets. For SNR calculation, the signal was defined as the mean over 3000 repetitions, while the noise was estimated as the standard deviation of the spectral noise floor, specifically within the 10–13 ppm and 15–20 ppm ranges. Figure S4: Scatter plot of the combined SNR for all voxels within the spherical phantom, comparing the self‐weighted method (red) and the 23Na‐based method (blue). SNRref represents the combined SNR obtained using the denoised 31P multi‐channel signal as the sensitivity reference. Figure S5: Monte Carlo simulation of a 16‐channel receiver array with randomized phases. The curve shows the L1 norm of the we [file MRM-95-1907-s001.docx]

^31^P MRSI coil combination using ^23^Na sensitivity information – Supplementary material

^1,2^Jiying Dai, ^1^Mark Gosselink, ^1^Zahra Shams, ^1^Martijn Froeling, ^1,3^Alexander J. E. Raaijmakers, ^1^Dennis W. J. Klomp

**Corresponding author**: Jiying Dai

**E-mail address**: jiyingdai@outlook.com

**Institutions**:

^1^University Medical Center Utrecht

^2^Tesla Dynamic Coils B.V.

^3^Eindhoven University of Technology

This document provides supplementary information for the study “^31^P MRSI coil combination using ^23^Na sensitivity information acquired with the same loop array at 7T”.

# SNR comparison when taking the 2^nd^ to the 50^th^ FID points for sensitivity approximation

In the main content of the manuscript, the in vivo data were treated by taking the 2^nd^ to the 5^th^ FID points for self-weighted sensitivity approximation. Taking more FID points will improve the SNR of the sensitivity information when the original SNR is low, resulting in a higher SNR for the combined signal. However, taking more FID points will increase the noise correlation between the sensitivity and the multi-channel signal. Consequently, the SNR will be over-estimated because the constructive interference of the correlated noise becomes more severe. Figure S1 presents the whole-volume SNR of Dataset 1 and 2 when taking the 2^nd^ to the 5^th^ and the 2^nd^ to the 50^th^ FID points, respectively, out of the total 256 FID points for sensitivity approximation. In addition to the self-weighted method and the ^23^Na-based method, we introduce a denoised-self-weighted method as a third reference. We see that the self-based method has a significantly increased apparent SNR when using more FID points, specially the PCr-based SNR. Taking more FID points barely changes the combined SNR for the ^23^Na-based method, meaning the ^23^Na-based method does not benefit from averaging more FID points, because the ^23^Na FID already has a very high SNR. The denoised-^31^P-based combination used the sensitivities approximated from PCA-denoised ^31^P signals^1^. We see that SNR_sum,PCr_ of the self-weighted method even surpasses that of the denoised-^31^P-weighted combination for both datasets, while SNR_sum,aATP_ does not show this pattern for either dataset. This strongly suggests that when more FID points are used for sensitivity approximation, the apparent improvement in PCr-based SNR observed with the self-weighted combination is primarily driven by overestimation, rather than by a genuine enhancement resulting from higher-SNR sensitivity information. The denoised-^31^P-based combination was not used as a reference in the manuscript due to its limitations. The observed SNR is already influenced by three factors: the intrinsic SNR of the sensitivity data, inaccuracies arising from the frequency offset between ^23^Na and ^31^P, and overestimation caused by constructive interference from correlated noise. Furthermore, the noise characteristics following PCA-based denoising have not been thoroughly investigated, making its use as a reliable reference method questionable in this context. In general, the denoising method does not eliminate noise entirely, nor does it do so in a linear manner. The extent of residual noise correlation between the approximated sensitivity and the signal remains unknown, making it difficult to assess the degree of overestimation. Consequently, we did not consider it suitable to serve as a gold standard in this study.


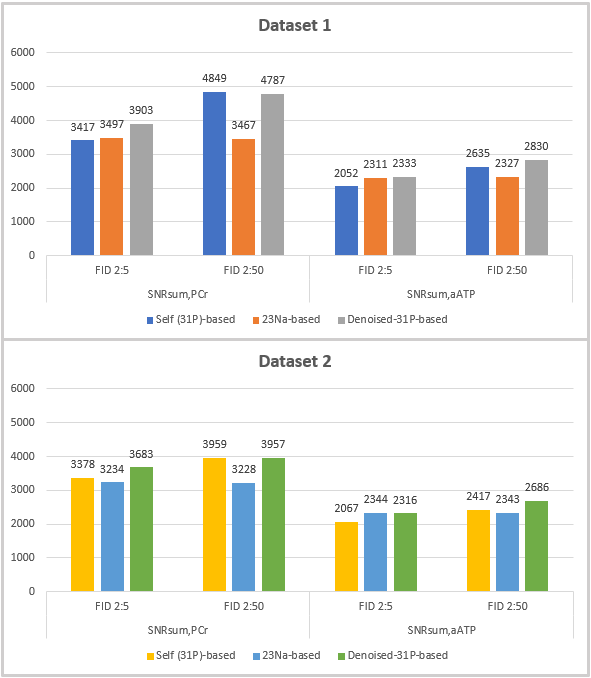


**Figure S1** The whole-volume SNR results for Datasets 1 and 2 are shown, comparing three coil combination methods: ^31^P (self)-weighted, ^23^Na-weighted, and denoised-^31^P-weighted. For each method, SNR values are presented using sensitivities approximated from the 2nd to 5th FID points and from the 2nd to 50th FID points, respectively. SNR is calculated in two ways: based on the PCr signal and based on the α-ATP signal.

# Additional picture of the hardware

This section presents the high-resolution image of the receiving loop array of the used coil (Figure 2D).


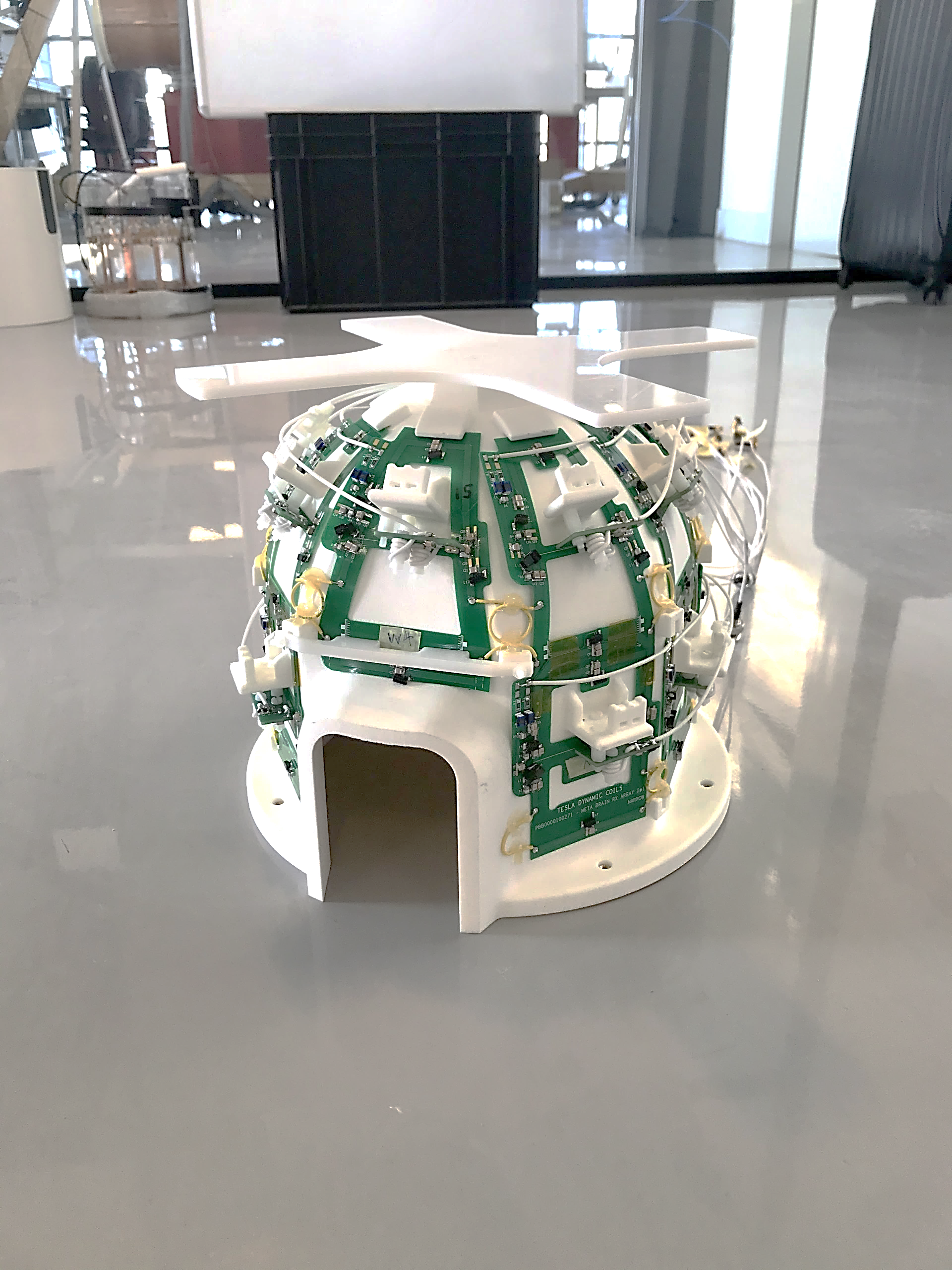


**Figure S2** High-resolution picture of the 16-channel ^31^P-^23^Na/^13^C receiver loop array of the used head coil.

# Safety constraints

The MR experiments have a constraint over average RF power. This section introduces the derivation of the power limit for the 8-channel ^1^H dipole array. We first performed EM simulations on DUKE to estimate the peak local SAR_10g_ and the head SAR with unit power per channel, with the dipole array driven in quadrature mode:

Peak local SAR_10g_ = 1.94 W/kg,

head SAR = 0.65 W/kg).

We then scaled the unit power to the SAR constraints according to IEC60601-2-33 (head SAR limit: 3.2 W/kg, local SAR limit: 10 W/kg):

Local SAR limit 10 W/kg allows: 10/1.94 ≈ 5.1 W/ch,

Head SAR limit 3.2 W/kg allows: 3.2/0.65 ≈ 4.9 W/ch.

We take the more conservative limit that is the head SAR limit, then applied 10% safety margin:

4.9/110% ≈ 4.4 W/ch.

At the installation of the used head coil, a B_1_^+^ mapping was performed after proper RF shimming to match the simulated B_1_^+^ to the reality. Then a calibration factor was applied to the average power limit for ^1^H scans. In this study, we required no SAR-demanding ^1^H scan, but only very brief scout images to localize the anatomy, thus not even close to the average power limit.

Same procedure was applied for the ^31^P bore coil, where the SAR is substantially lower as a large volume coil at a much lower frequency (120.68 MHz) was used.

For ^23^Na, we took the most conservative strategy. Assuming all power out of the ^23^Na RF amplifier is deposited to the subject (human head, 5 kg) with no loss to the environment nor the electronics, a total of 16 W average power is allowed (head SAR limit multiplied by the head mass). We took this 16 W as the average power limit. In reality, a significant amount of the power would be lost in the transmit chain or would dissipate in the environment. However, as ^23^Na has a very high SNR in vivo (highest among all the X-nuclei), we do not need high RF power of ^23^Na in this application. A non-optimized ^23^Na scan at ^31^P resolution did easily provide 10 times the SNR of the ^31^P scan, that is beyond sufficient for sensitivity approximation.

# Extensive Monte-Carlo-based analysis

At this stage, we are not yet able to disentangle the SNR overestimation caused by complex noise from the combined results. To clearly illustrate the presence of such overestimation, we present in this section a series of Monte Carlo-based analyses. Using this model, we also explore the limitations of the ^23^Na-based method. While the results presented in this study are encouraging, it remains of interest to evaluate how far the ^23^Na-based combination can outperform—or fall behind—the self-weighted approach under different conditions.

For this simulation, the acquired ^23^Na data were assumed to have a fixed post-combination SNR of approximately 150, which was estimated according to Dataset 1 and 2. The resulting estimations are shown in Figure S3.

The x-axis represents the true total SNR, selected to reflect the feasible range for in vivo ^31^P MRSI. The grey line (slope = 1) marks the theoretical upper limit of the combined SNR, which equals the true total SNR. The solid red line shows the self-weighted combination where the coil sensitivities were approximated from the average of the second to fifth FID points—a commonly used approach. The red dashed line represents an extreme case where sensitivities are estimated by averaging all 256 FID points, effectively approximating a root-sum-of-squares combination.

When observing the PCr signal (Figure S3A), both self-weighted approaches exhibit overestimation. In the 4-FID scenario, this bias is clearly visible in the low-SNR regime (true SNR < 10). In the full-FID case, the improved sensitivity SNR (benefitted from averaging a larger number of FID points) significantly boosts the combined SNR—but at the cost of severe overestimation bias. This overestimation is not limited to low-SNR conditions but persists even at relatively high SNR levels—the red dashed line exceeds the grey line even when the measured (combined) SNR is above 20. On the other hand, even at a true SNR level of zero, the reconstruction still yields a finite SNR for PCr. This occurs because the noise contributions from different channels at the PCr frequency are constructively combined.

The solid blue line in Figure S3 corresponds to the ^23^Na-based combination, using the second to fifth FID points of ^23^Na data with realistic in vivo SNR. The ^23^Na-based method demonstrates consistent SNR improvement over the self-weighted method, theoretically. In practice, we do not see such dominant outperformance of the ^23^Na-based method. Our in-vivo measurements show that for realistic SNR values, the ^23^Na-based method does outperform the self-weighted method but not by this much. Therefore, we anticipate the existence of an SNR threshold beyond which the self-weighted method becomes superior. For example, in Figure 8, no clear improvement from the ^23^Na-based approach is observed when the PCr-based SNR exceeds approximately 6. Based on this, we hypothesize that the blue dashed line represents a more realistic estimate of the ^23^Na-based method’s performance in our specific experimental context. This deviation is likely due to a residual mismatch between the ^23^Na and ^31^P sensitivity maps. Although EM simulations suggest sufficient similarity in their B_1_^-^ fields, amplitude scaling discrepancies along the receive chain, which we find difficult to characterize, may still exist.

Nonetheless, ^31^P MRSI in the brain already represents a relatively high-SNR scenario. This suggests that most other in vivo application would fall to the left of our ‘hypothesized’ threshold, where the ^23^Na-based method demonstrates superior performance.


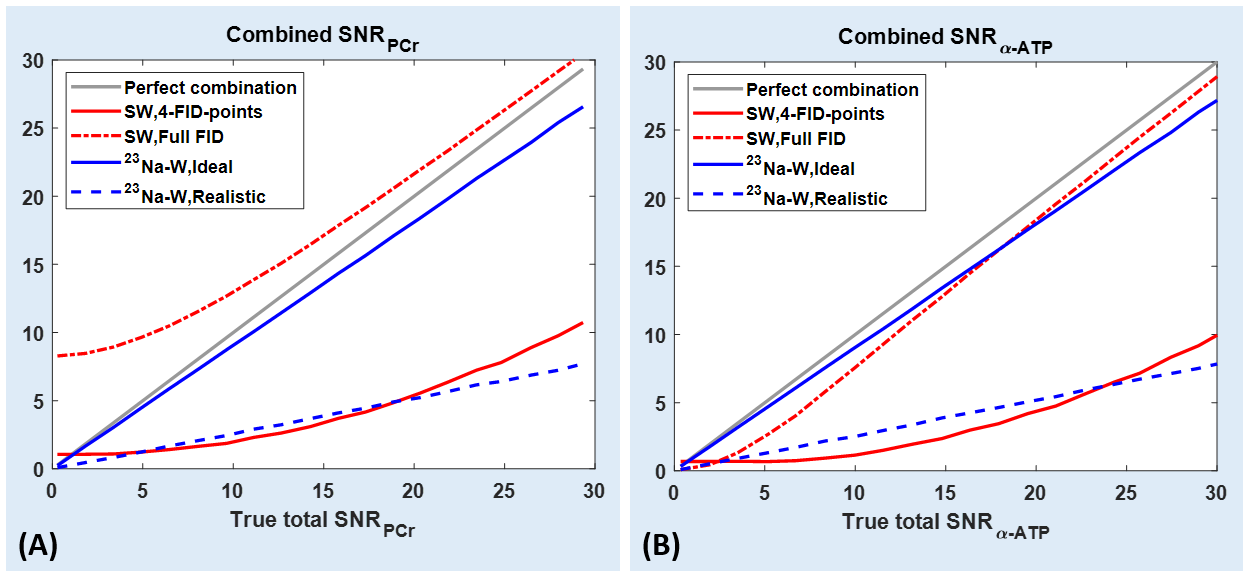


**Figure S3** Monte Carlo-based SNR estimations performed for the 15-channel coil combination using both the self-weighted and the ^23^Na-based methods, across a range of intrinsic total SNR values. Additionally, a self-weighted method using the full FID (all 256 FID points) average for sensitivity approximation is included, demonstrating the extent of overestimation in this extreme scenario. The ‘Perfect combination’ represents the theoretical upper limit of the combined SNR; any value above it indicates an overestimation bias. The SNR was evaluated based on the PCr peak (A) and the α-ATP peak (B), respectively. The synthetic ³¹P spectrum shown in Figure 6A was used as the input. The SNR ratio between the ^23^Na data (used for sensitivity approximation) and the ^31^P data was aligned with values observed in the presented in vivo datasets. For SNR calculation, the signal was defined as the mean over 3000 repetitions, while the noise was estimated as the standard deviation of the spectral noise floor, specifically within the 10–13 ppm and 15–20 ppm ranges.

# Verification on phantom

We further validated the Monte Carlo simulation using phantom experiments. The experiments were carried out using the same RF coil setup as the in vivo experiments. An 18-cm diameter spherical phantom containing 9 g/L H2KPO4 and 4g/L NaCl was used, and the scan protocol is summarized in Table S1.

|  | Sequence | Resolution | Voxel number | TR/TE (ms/ms) | FA (degree) | NSA | Bandwidth (Hz) | Number of samples |
| --- | --- | --- | --- | --- | --- | --- | --- | --- |
| ^23^Na | 3D FID | 2×2×2 cm^3^ | 11×12×10 | 60/0.55 | 12 | 20 | 5000 | 256 |
| ^31^P | 3D FID | 2×2×2 cm^3^ | 11×12×10 | 60/0.5 | 12 | 20 | 5000 | 256 |

**Table S1** Scan parameters of the phantom experiment.

Since phantom data exhibits a substantially higher ^31^P SNR than in vivo data, we added Gaussian noise to reduce it to the regime of interest. The ^23^Na data was left unchanged. Unlike in the simulation, where channel combination with noise-free sensitivities is available as an ideal reference, the most ideal sensitivity achievable in the phantom experiment was the denoised^1^ FID of the original high-SNR ^31^P phantom data prior to noise addition.

Accordingly, we combined the “noised” phantom data using three different sensitivities: (1) the semi-ideal sensitivity described above, (2) the average of the second to fifth FID points of the to-be-combined ^31^P data, and (3) the average of the second to fifth FID points of the ^23^Na data. The combined SNRs obtained from the second and third methods are presented as a scatter plot in Figure S4, with the first method serving as the x-axis reference (SNR_ref_ ​). Each point corresponds to the post-coil-combination SNR of a voxel within the phantom. It should be noted that the x-axis here is not equivalent to that in Figure S3; therefore, the absolute y-to-x slopes cannot be directly compared between Figures S3 and S4.

Figure S4 closely resembles the red solid and blue dashed lines shown in Figure S3. When SNR_ref_ falls below 5, the self-weighted method is dominated by over-estimation rather than the true SNR, producing a non-zero plateau. In contrast, the ^23^Na-based method exhibits a nearly linear trend in general. Furthermore, when SNR_ref_ ​ is below approximately 20, the ^23^Na-based approach outperforms the self-weighted method. The MATLAB code and the data are shared on GitHub^2^.


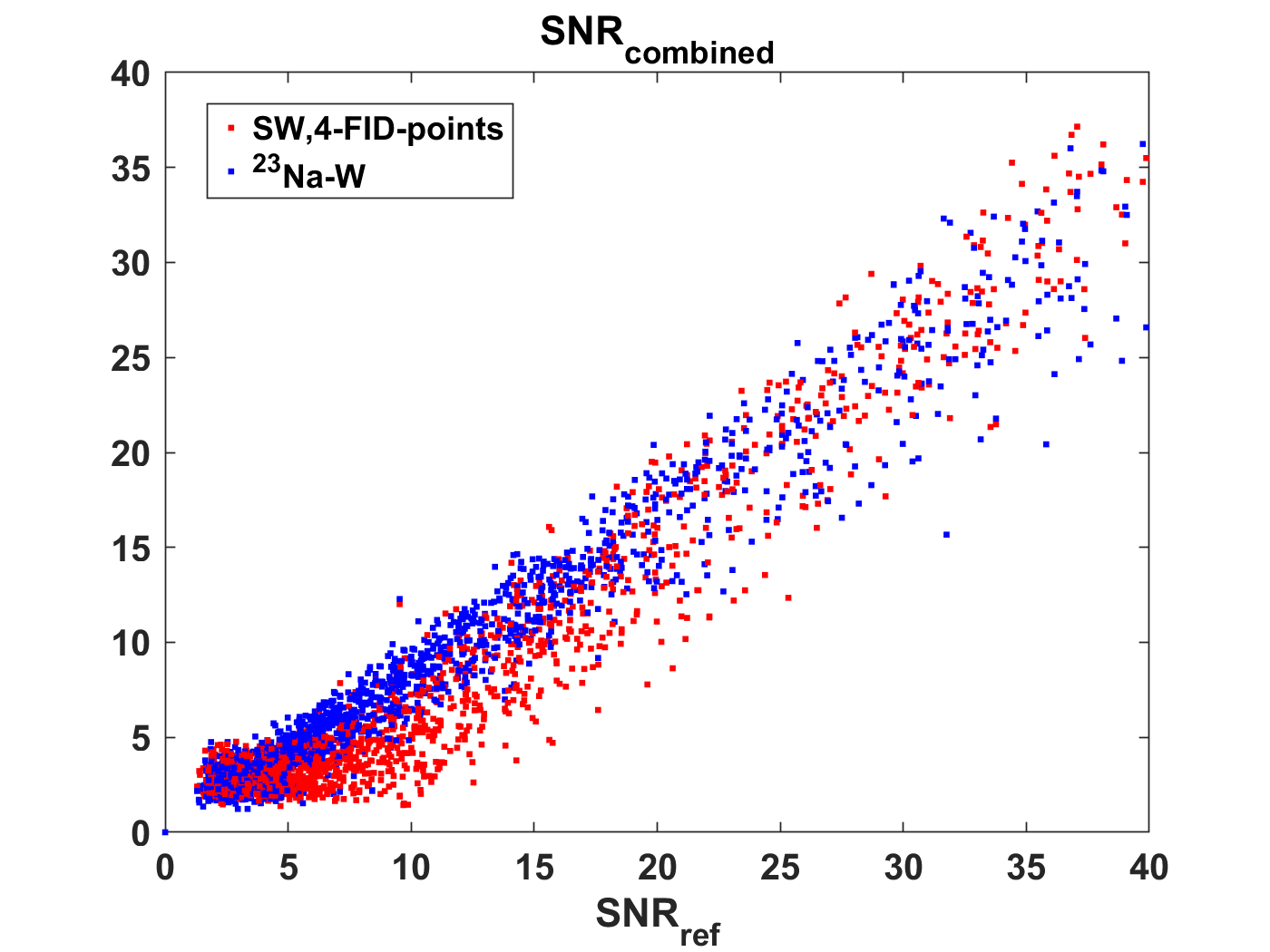


**Figure S4** Scatter plot of the combined SNR for all voxels within the spherical phantom, comparing the self-weighted method (red) and the ^23^Na-based method (blue). SNR_ref_ ​represents the combined SNR obtained using the denoised ^31^P multi-channel signal as the sensitivity reference.

# Norm of the weight vector

In the Discussion section, we stated that the sensitivity SNR directly influences the SNR of the combined signal. This section outlines the theoretical basis for that statement. In Roemer’s framework, the combination weight vector is expressed as $\frac{\mathbf{R}^{-\frac{1}{2}}\mathbf{b}}{\sqrt{\mathbf{b}^{H} \mathbf{R}^{-1}\mathbf{b}}}$. After noise decorrelation, the noise correlation matrix becomes an identity matrix, simplifying the weight vector to $\frac{\mathbf{b}}{\sqrt{\mathbf{b}^{H}\mathbf{b}}}$, where sensitivity $\mathbf{b}$ is an N_ch_-by-1 vector. When the sensitivity is approximated as the average of the first five FID points of an N_ch_-channel noisy signal, each element of $\mathbf{b}$ then follows a complex Gaussian distribution. Consequently, the normalized weight vector $\frac{\mathbf{b}}{\sqrt{\mathbf{b}^{H}\mathbf{b}}}$ follows a Kent distribution. The norm of this vector determines the SNR of the combined signal. For noise-free $\mathbf{b}$, the $L^{1}$ norm equals $\sqrt{N_{\mathrm{ch}}}$, meaning the combination fully preserves the available SNR. Conversely, if $\mathbf{b}$ has 0 SNR, the norm approaches 0, and no SNR is gained from the combination.

The precise relationship between the weight vector norm and the per-channel SNR is illustrated in Figure S5. This relationship was derived from Monte Carlo simulations, in which sensitivities of 16 channels with randomized phases were synthesized with added noise. The $L^{1}$ norm was computed as the average of 10^4^ repetitions across a per-channel SNR range of 0 to 22. For in vivo ^31^P MRSI, the per-channel SNR is usually below 5 for all metabolite peaks.


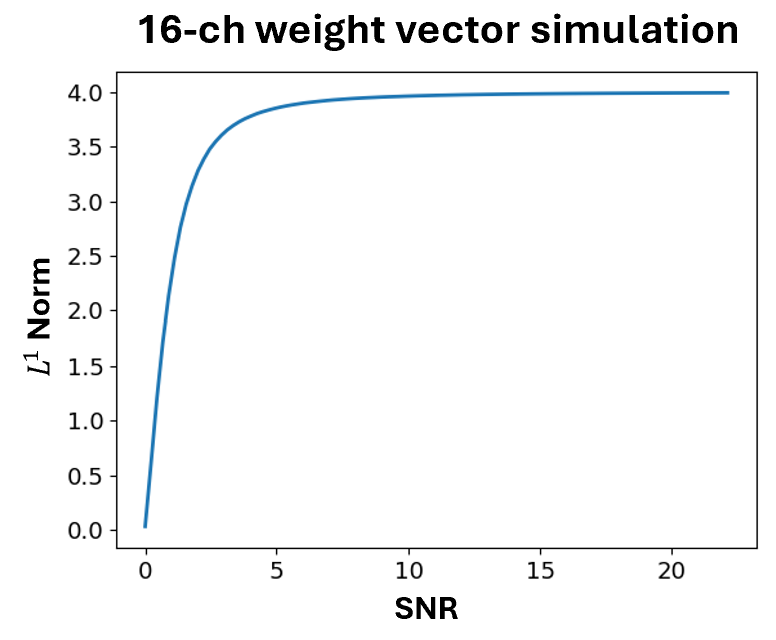


**Figure S5** Monte Carlo simulation of a 16-channel receiver array with randomized phases. The curve shows the $L^{1}$ norm of the weight vector as a function of per-channel SNR (0–22), averaged over 10^4^ repetitions.

# References

1. Froeling M, Prompers JJ, Klomp DWJ, van der Velden TA. PCA denoising and Wiener deconvolution of 31P 3D CSI data to enhance effective SNR and improve point spread function. *Magn Reson Med*. 2021;85(6):2992-3009.

2. Dai J. GitHub - JiyingDai/Na4Pho: Codes for the Na4Pho project. Preprint posted online 2024. https://github.com/JiyingDai/Na4Pho
